# Assessing biodiversity of a freshwater benthic macroinvertebrate community through non-destructive environmental barcoding of DNA from preservative ethanol

**DOI:** 10.1186/1472-6785-12-28

**Published:** 2012-12-23

**Authors:** Mehrdad Hajibabaei, Jennifer L Spall, Shadi Shokralla, Steven van Konynenburg

**Affiliations:** 1 Biodiversity Institute of Ontario, Department of Integrative Biology, University of Guelph, Guelph, Ontario, N1G 2W1, Canada

## Abstract

**Background:**

Characterizing biodiversity in a habitat or in targeted taxonomically or socioeconomically important groups remains a challenge. Standard DNA-based biodiversity identification tools such as DNA barcoding coupled with high-throughput Next-Generation Sequencing (NGS) technologies are rapidly changing the landscape of biodiversity analysis by targeting various habitats and a wide array of organisms. However, effective use of these technological advances requires optimized protocols and benchmarking against traditional tools. Here we investigate the use of commonly used preservative ethanol as a non-destructive and inexpensive source of DNA for NGS biodiversity analysis of benthic macroinvertebrates. We used the preservative ethanol added to field collected organisms (live sorted bulk benthic samples) as a source of community DNA for NGS environmental barcoding. We directly compare this approach with a DNA barcode library generated using Sanger sequencing of all individuals separated from abenthic sample as well as with NGS environmental barcoding of DNA extracted from mixed/homogenized tissue specimens of the same benthic sample. We also evaluate a multiplex PCR strategy, as compared to commonly used single amplicon workflow, using three newly designed primer sets targeting a wide array of benthic macroinvertebrate taxa.

**Results:**

Our results indicate the effectiveness of ethanol-based DNA in providing sequence information from 87% of taxa identified individually from mixture as compared to 89% in conventional tissue extracted DNA. Missing taxa in both DNA sources were from species with the lowest abundance (e.g. 1 individual) in the benthic mixture. Interestingly, we achieved 100% detection for taxa represented with more than 1% individuals in the mixture in both sources of DNA. Our multiplex amplification regime increased the detection as compared to any single primer set indicating the usefulness of using multiple primer sets in initial amplification of target genes.

**Conclusions:**

Although NGS approaches have significantly increased the potential of using DNA information in biodiversity analysis, robust methods are needed to provide reliable data and alleviate sample-processing bottlenecks. Here we coupled non-destructive DNA access and a multiplex PCR approach in NGS environmental barcoding for effective data generation from benthic live-sorted samples collected in bulk and preserved in ethanol. Our study provides a possible solution to sampling and vouchering challenges in using benthic samples through next-generation environmental barcoding and facilitates wider utility of DNA information, especially species-specific DNA barcodes, in ecological and environmental studies and real-world applications such as biomonitoring programs.

## Background

Next-generation sequencing (NGS) technologies are rapidly increasing the applicability of genomics approaches in biological sciences. Aside from the sheer volume of sequence information obtained from NGS devices, the massively parallelized capacity of these machines is a significant advantage for the analysis of mixtures of DNA fragments. In PCR-based applications of NGS technologies amplicons are generated from target genes, often from multiple template genomes, and are sequenced in parallel without the need for separating target organisms or their DNA through sample sorting or cloning [1]. For example, microbial ecologists have taken advantage of NGS technologies in amplicon-based (marker genes) metagenomic studies such as the analysis of 16S rDNA for prokaryotic biodiversity assessments in various ecological settings [2]. Similar approaches have been developed for many situations where multi-template (environmental) samples are targets of investigations [3].

Environmental barcoding [4] seeks to gain sequence information for standardized species-specific DNA markers—DNA barcodes—directly from environmental samples such as soil, water, air, benthos or gut contents of various organisms [4]. This approach has the potential to dramatically increase the applicability of DNA barcoding in situations where rapid assessment of biodiversity at high resolution (e.g. species-level) is required at a wide spatiotemporal scale or in places where access to individual specimens is impossible or impractical. For example, environmental assessment through biomonitoring relies on biodiversity patterns of bioindicator (sentinel) species such as benthic macroinvertebrates, often at larval stage. However, due to difficulties in robust species-level identification of target groups, biomonitoring programs are faced with an identification bottleneck that can then lead to difficulties in implementing these programs [5]. We have recently demonstrated the potential of using NGS-based environmental barcoding in identifying species of fresh water benthos [4]. This approach has triggered a wholly new biomonitoring paradigm--Biomonitoring 2.0--for environmental assessment [6].

An important concern in the analysis of environmental samples is obtaining DNA templates from all target organisms in the mixed sample. Methods have been developed to extract and purify DNA from environmental samples such as soil or water. However, for bulk material such as benthos--obtained using kick nets--orpassively sampled arthropods collected in a Malaise trap, DNA extraction often requires homogenizing the biomass from all organisms and then performing a standard DNA extraction protocol on this homogenized slurry. Although this approach has been effective in gaining DNA from organisms in the mixture, it results in loss of all individual specimens, thereby rendering any subsequent analysis on these individuals impossible. Recently, we demonstrated that ethanol, commonly used as a preservative medium for storing specimens, contains DNA of stored organism and that this “free DNA” can be directly used for downstream amplification and sequencing without the need for conventional DNA extraction approaches [7]. Although we have shown the utility of this approach in individual specimens stored in ethanol, it is not known whether ethanol-based free DNA of many different taxa, with various biomasses, in a mixed environmental sample such as benthos, could be sequenced in an NGS workflow.

Another important concern in the use of NGS for analysis of environmental samples is the issue of bias in multi-template PCR amplification [8]. Current workflows often require PCR amplification of target templates from mixed samples, which can result in differential amplification of sequences from some species, leading to qualitative and quantitative biases in sequence representation from target organisms in the mixture. In other words, because of PCR bias, some species may not be amplified and sequenced while others may be amplified and sequenced in excess. This can obscure identity and abundance measures from bulk environmental samples. Although modified amplification regimes have been developed for offsetting the effect of PCR bias [8] and methods based on direct sequencing of DNA (without the need for PCR amplification) are on the horizon [9], PCR amplification bias remains an important issue. This problem is especially important when environmental samples are used for surveillance and monitoring applications where comparative analysis of biodiversity should be performed objectively and reproducibly [6].

Here we introduce an enhanced approach for environmental barcoding, which will aid sampling, DNA extraction and PCR steps in biodiversity analysis of benthic macroinvertebrate taxa commonly used for biomonitoring applications. We firstly incorporate a non-destructive sample preparation approach by using preservative media (ethanol) as the source of target DNA (hereafter referred to as ethanol-based DNA) and compare it with a conventional tissue-based DNA extraction (hereafter referred to as tissue-DNA). Secondly, to increase the recovery of species’ DNA barcode sequences in bulk environmental samples and to offset specific primer-binding biases, we introduce a multiplex PCR approach targeting multiple amplicons within the standard cytochrome c oxidase 1 (COI) DNA barcode region. We develop and test three wide-range primer sets for NGS analysis. To show the utility of this approach, we test it in parallel with Sanger sequencing individual specimens from a typical biomonitoring benthic sample containing several groups of macroinvertebrates.

## Results

### Sanger sequencing analysis of individual specimens

**Figure 1:**
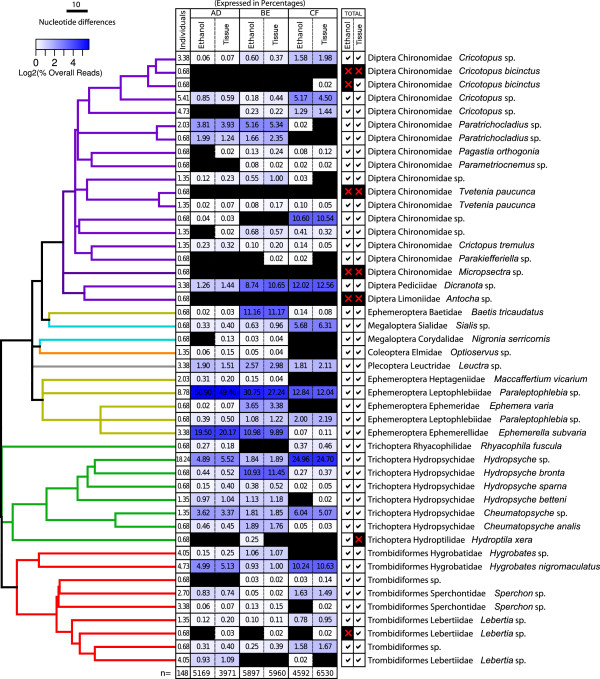
**Taxonomic composition of the benthic sample analysed from tissue-DNA and ethanol-based DNA.** The tree is assembled through neighbor-joining analysis of COI DNA barcodes obtained in Sanger sequencing of individual tissue samples and color-coded to visualize different orders. The first column, next to branches, represents percentage of individuals (from a total of 148) for each OTU as identified through Sanger sequencing DNA barcoding. Six subsequent columns represent percentage of sequence reads for taxa in 454 pyrosequencing analysis using the two sources of DNA from mixture (ethanol-based DNA and tissue-DNA) by three primer sets (AD, BE, CF). The final two columns represent total evidence as pass/fail for each taxa using combined data from three primer sets for ethanol-based DNA and tissue-DNA. Cells are color-coded as a heat map based on percentage 454 reads to facilitate visualization of quantitative trends. Black cells represent negative results (no sequence was obtained).

Our first analysis aimed at assembling a DNA barcode sequence library from all individuals present in our benthic sample. Using a small (i.e. 2 mm) tissue sample (usually a leg), from each larvae, we were able to obtain a standard COI DNA barcode sequence from 98.7% (148/150) of individuals present in the benthic sample. These Sanger sequenced individuals formed 46 OTUs in a neighbor-joining tree analysis of COI barcodes. When these taxa were identified to lowest annotated taxonomic unit 36.9% (17 OTUs) of them matched DNA barcode sequences of known species and another 50% (23 OTUs) matched a DNA barcode identified at genus level. The remaining 13.1% matched a sequence at family or order levels (Figure 1). The most diverse group in the analysis of Sanger sequences was true flies (19 OTUs; 41.3%), followed by mites (9 OTUs; 19.6%) and caddisflies (8 OTUs; 17.4%).

The most abundant OTU in the environmental sample was caddisflies (Trichoptera) identified to genus *Hydropsyche* and representing 18.2% of Sanger sequenced individuals, followed by mayflies (Ephemeroptera) from genus *Paraleptophlebia* representing 8.78% of individuals. All other species had lower than 5% abundance in Sanger sequencing analysis (Figure 1).

### Sequence recovery from ethanol-based DNA

The NGS analysis of ethanol-based DNA, provided sequences from 87% of OTUs found in the assembled Sanger DNA barcode library. In comparison, 89% of OTUs were obtained through analysis of tissue-DNA extracted from the homogenized mixture of larvae. Both approaches missed the same four OTUs but ethanol-based DNA also failed to detect an additional two. One of these belonged to a midge species *Cricotopus bicinctus*, was only detected by one primer set and was represented by a small percentage of 454 sequence reads (see below) in the tissue-DNA analysis. This species had two OTUs in the Sanger sequence library. One of these was missed in both ethanol-based DNA and tissue-DNA analysis (Figure 1). The other missing OTU in ethanol-based DNA analysis was a mite from the genus *Lebertia*. On the other hand, ethanol-based DNA analysis detected the caddisfly species *Hydroptila xera*, which was not detected in tissue-DNA. Interestingly, all missing OTUs in both ethanol-based DNA analysis and the extracted tissue-DNA analysis belonged to taxa with the lowest abundance (i.e. 1 individual) in the environmental sample tested (Figure 1).

### Multiplex PCR versus single primer set analysis

In both ethanol-based DNA and tissue-DNA, single primer set analysis detected fewer OTUs than multiplex analysis. Table 1 summarizes our observations. Although we did not find substantial differences between each primer set, primer set BE, which produced a 224 bp fragment of COI, was somewhat superior in detecting species in both ethanol-based DNA and tissue-DNA. Primer set CF (197 bp amplicon), on the other hand, was the least efficient and detected only 67.4% of the OTUs in both sources of DNA , although the composition of missing taxa varied between the two (Figure 1).

**Table 1:** Percentage detection of taxa in environmental barcoding analysis of two sources of DNA using three PCR primer sets

**Primer set**	**AD**	**BE**	**CF**	**AD** + **BE**	**AD** + **CF**	**BE** + **CF**	**ALL**
**ETHANOL**-**DNA**	69.57	78.26	67.39	84.78	82.61	86.96	86.90
**TISSUE**-**DNA**	78.26	80.43	67.39	86.96	86.96	86.96	89.10
**ETHANOL** + **TISSUE**	78.26	82.61	76.09	89.13	89.13	91.30	91.30
**ETHANOL** >**1**%	91.30	95.65	78.26	100	100	100	100
**TISSUE** >**1**%	95.65	95.65	73.91	100	100	95.65	100

>1% denotes taxa with more than 1% individuals in the benthic mixture tested here.

Percentages are calculated based on a total of 148 OTUs obtained in Sanger-based DNA barcode library.

ETHANOL = ethanol-based DNA; TISSUE = extracted tissue-DNA.

When we compared combinations of two primer sets (two amplicons), detection of taxa increased substantially for all two-amplicon combinations (Table 1). Finally, a combination of all three amplicons improved detection of taxa in tissue-DNA (only by 2%) but not in ethanol-based DNA analysis. Although, as stated earlier, in the analysis of ethanol-based DNA, 2% fewer taxa were detected as compared to tissue-DNA (see below).

### Primers versus DNA source and total evidence

We combined data obtained from ethanol-based DNA and tissue-DNA to see the effect of using both sources of DNA in detection capability (Table 1). Using DNA from two sources in single amplicon analyses improved detection in two primer sets (BE and CF). Primer set BE showed the highest detection for combined ethanol-based DNA and tissue-DNA (87%). This number is 7% higher than when tissue-DNA was analysed alone using this primer set. Subsequently, we compared the two-primer set combinations (two amplicons) in both ethanol-based DNA and tissue-DNA. These combinations improved the detection and one of them (BE and CF amplicons) provided sequence evidence for 91.3% of taxa in the Sanger library (Table 1). When all combinations of primers in two sources of DNA were combined (total evidence), we were able to still detect 91.3% of taxa in our Sanger library (Table 1). Missing taxa in this total analysis belonged to taxa represented by 1 individual (lowest abundance) (Figure 1).

**Figure 2:**
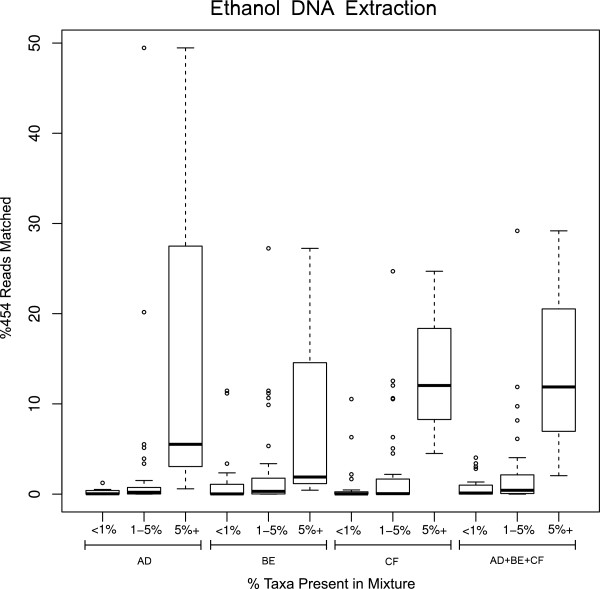
**Quantitative analysis of taxa based on 454 sequences as represented in box plots of percentage of sequence reads obtained from ethanol**-**based DNA in different primer sets and their combined data.** Taxa are binned in three abundance groups (less than 1%, 1–5%, and more than 5%) based on their number of individuals in the benthic sample analysed.

**Figure 3:**
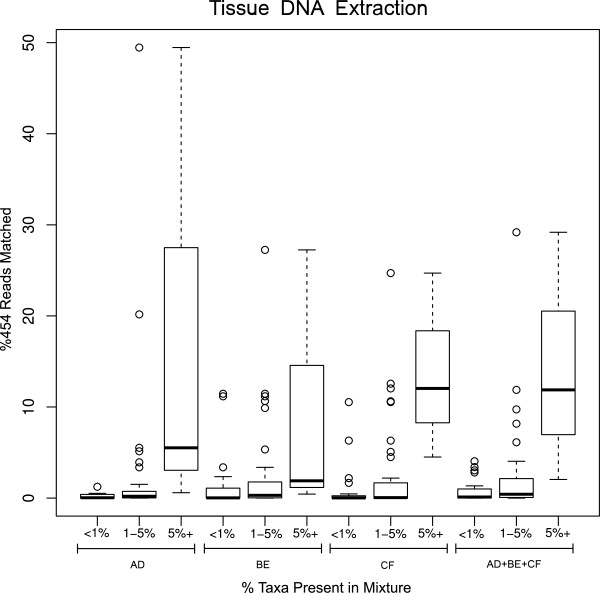
**Quantitative analysis of taxa based on 454 sequences as represented in box plots of percentage of sequence reads obtained from tissue**-**DNA in different primer sets and their combined data.** Taxa are binned in three abundance groups (less than 1%, 1–5%, and more than 5%) based on their number of individuals in the benthic sample analysed

We also compared different primer sets for their efficiency in detecting species quantitatively. In other words, we simply calculated the percentage of taxa with more than 1% abundance in our benthic mixture that were detected by each primer set (Table 1). Interestingly, although single amplicon analysis of AD and BE primer sets detected 95% of abundant taxa (more than 1% individuals) all but one two-amplicon combinations were able to detect 100% of these taxa in the mixture (Table 1; Figure 1). Moreover, to demonstrate quantitative trends of our benthic community analysis through 454 pyrosequencing, we plotted percentage of reads obtained from each of three primer sets and their combination in ethanol-based DNA analysis (Figure 2) and in tissue-DNA analysis (Figure 3). It is clear that taxa with more individuals show a general trend towards obtaining more 454 sequence reads, however, there are outliers in each primer-set that go against this general trend (Figures 2 and 3).

**Figure 4:**
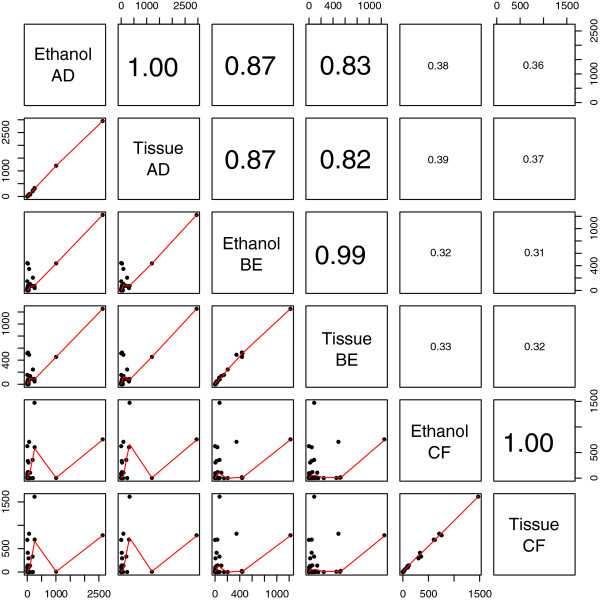
**Correlation analyses of environmental barcoding using different sources of DNA and primer sets as shown in number of 454 sequence reads matching each taxon from reference Sanger library.** Graphs are represented below diagonal squares. In each graph number of 454 reads are plotted for two different 454 experiments comparing sources of DNA (ethanol-based DNA versus tissue-DNA) and primer sets used. Diagonal squares represent labels for X and Y-axis in columns and rows. For example, the first graph represents data from ethanol-based DNA using primer set AD in X-axis and tissue-DNA using primer set AD in Y-axis. R^2^ values for each graph are represented above diagonal squares and correspond to mirroring graphs below diagonal squares. For example, the analysis of ethanol-based DNA versus tissue-DNA in primer set AD is supported by an R^2^ value of 1. Font size corresponds to higher R^2^ values.

Further to our investigation to evaluate taxonomic detection (see above), we performed a series of correlation analyses to understand primer behavior as well as uniformity of results among different comparisons in ethanol-based DNA and tissue-DNA reactions. We used the proportion of reads obtained for each OTU for calculating correlations (Figure 4). Each primer set showed an almost perfect correlation when compared between ethanol-based DNA and tissue-DNA (Figure 4). Other strong correlations belonged to primer sets BE and AD in both ethanol-based DNA and tissue-DNA, as well as between the two different DNA templates (Figure 4). On the other hand, sequences obtained from primer set CF did not correlate strongly with the other two primer sets in either ethanol-based DNA or tissue-DNA comparisons (Figure 4).

### Recovery of sequences from taxa absent in Sanger library

A small number of NGS reads (Min 5 and Max 95 in different primer sets and DNA sources) matched sequences of 16 OTUs that were not originally in our assembled Sanger library (Additional file 1: Table S1). Interestingly, 81% of these OTUs belonged to taxonomic groups commonly found in benthic samples. As such, NGS analyses revealed more biodiversity as compared to Sanger analyses of single specimens. The remaining sequences matched human (14 reads) and poplar (3 reads) in analysis of ethanol-based DNA using primer set BE, and dog (1 read) in analysis of tissue-DNA using primer set AD (Additional file 1: Table S1).

## Discussion

Although NGS approaches have dramatically increased the capacity of genomics applications in biodiversity science, these applications have so far focused on discovering biota rather than monitoring their changes. As such, issues related to efficiency, repeatability and robustness have not been fully explored. A biomonitoring application based on NGS analysis of communities [6] requires robustness and reproducibility. This work was an attempt in demonstrating the utility of ethanol as a source of DNA in a multiplex PCR approach for NGS-based environmental barcoding.

Our previous work has demonstrated the utility of leaked or ethanol-based DNA from preservative ethanol for direct PCR amplification and subsequent Sanger sequencing of single specimens [7]. Here we extended this approach for a mixed community of benthic taxa collected using standard aquatic biomonitoring approaches and analyzed using an NGS workflow. Although this approach is somewhat similar to using environmental DNA (e-DNA), typical e-DNA does not involve collecting and storing organisms even as a mixture that we used here [10]. In fact, we take advantage of ethanol as a widely used preservative medium to access DNA from organisms nondestructively. Our approach will leave physical samples (i.e. individual larval samples) intact and accessible for subsequent molecular or morphological examinations, if needed. Additionally, this ethanol-based DNA does not require any DNA extraction procedure and can be directly amplified and sequenced, reducing the cost and time required for analysis.

The fact that ethanol used for accessing DNA contains a mixture of taxa with different sizes and abundances, does not seem to affect recovery of their sequences from ethanol-based DNA, as compared to tissue-DNA. We were able to recover many organisms that were only represented in small quantities in the mixture through the analysis of their DNA from ethanol. Missing taxa in both DNA sources were mainly from species with low abundance in our benthic sample and taxa with more than 1% were seldom missed. This pattern is strongly reflected in high correlation between the number of reads from two sources of DNA using different primer sets and leads us to deduce that primers bind to each species in the mixture with the same efficiency between two different sources of DNA. In other words, ethanol-based DNA seems to be a good replacement for DNA extracted from tissue mixtures, opening the door to a significant advantage for nondestructive analysis of benthic samples preserved in ethanol and can potentially be used in similar situations where a group of organisms are stored in a preservative media (see below).

Our results from both tissue extracted DNA and ethanol-based DNA suggest the presence of taxa not originally sequenced in our Sanger-based DNA barcode analysis. While two individual larval samples did not produce Sanger DNA barcodes in our analysis, 14 benthic taxa were detected that were not present as individuals in our mixture. Since our analysis was based on a single sampling event using all precautions to avoid possible contamination we can potentially link the presence of residual DNA to carry over through the organisms present in the larval community we sampled. This is in line with earlier observations [4].

Our study only included live field-sorted larval mixtures (a common sampling approach used for aquatic biomonitoring) and needs to be verified in other types of mixed samples such as Malaise traps (used for terrestrial arthropods), pitfall traps (used for soil arthropods) and different types of light traps (used for insects). A somewhat comparable approach for accessing extracellular DNA has been advocated for soil analysis, although it uses a phosphate buffer followed by a DNA extraction protocol [11].

Oligonucleotide primers are an integral factor in PCR-based NGS analysis, yet there has not been much attention to empirical optimization and testing of primers for specific genes commonly used in NGS analysis. Conversely, genes have mainly been selected based on availability of conserved binding sites [12]. It is, however, well known that differential primer binding can lead to quantitative and qualitative bias in taxa recovered from a mixed environmental sample even when primer-binding sites are conserved in a wide range of taxa [8]. Consequently, to offset the effect of biases in primer binding, in this study we used three newly developed primer sets for targeting benthic macroinvertebrate taxa commonly used in biomonitoring. Our results further show the effect of primers in taxa recovery but our combined approach does seem to alleviate PCR primer binding bias as we were able to recover up to 90% of all taxa and 100% of taxa with higher than 1% abundance, even though we used a relatively small sequencing throughput for this analysis (e.g. 1/16 of a 454FLX Titanium run for each DNA template from mixture). An increase in sequencing depth in a PCR regime using multiple primers may reduce or eliminate false negatives, especially for species with a smaller number of individuals or lower biomass in a sample. Although our study did not aim at quantitative analysis of taxa, we see high recovery (more sequence reads) from multiple primers in species that are represented by more individuals in the mixture (Figures 2 and 3). A similar pattern was observed in our previous study using only a single shorter mini-barcode fragment [4]. These early observations may provide the basis for developing a multiplex PCR regime for quantitative analysis of abundance as measured through biomass and considering gene copy number and other factors in different taxa. Measuring species abundance has been a key metric in traditional biomonitoring and ecological investigations but a recent study provides evidence that presence or absence alone can be used as a data source for a benthic response index used for biomonitoring [13].

## Conclusions

The difficulty in large-scale spatiotemporal analysis of biodiversity has resulted in bottlenecks in executing biomonitoring programs and several other types of ecological and environmental investigations [6]. Next generation sequencing technologies coupled with high-resolution marker genes such as species-specific DNA barcodes--environmental barcoding--can address data rarity and low taxonomic resolution often crippling biodiversity analysis. Our study, additionally, allows nondestructive environmental barcoding analysis of benthic samples preserved in ethanol. If our observations are confirmed in a wider variety of biological specimens that are regularly collected in “ethanol jars”, this approach can pave the way for different applications, which require scanning the contents of these specimens for target taxa such as pests, pathogens, vectors, and rare or endangered organisms. The nondestructive nature of the ethanol-based analysis allows for subsequent thorough investigations to potentially separate and study these targeted taxa using different molecular or morphological techniques.

Approaches such as environmental barcoding are relatively new and require additional scrutiny to make them suitable for large-scale studies and real-world applications. Here we show the utility of ethanol-based DNA in simplifying sample preparation and alleviating destructive analysis of ethanol-preserved organisms. This procedure also allows preservation of specimens for further scrutiny. We also introduced a new multiplex PCR approach to neutralize specific primer-binding biases, thereby allowing the recovery of more taxa. We recognize that multiple samplings are required for comprehensive analysis of biota in an ecosystem through NGS. The availability of NGS devices with higher throughput and lower costs per analysis will facilitate generating sample sizes required for sound statistical analysis of NGS results [9]. We note, however, that our entire NGS data sets presented here were assembled using only two lanes of a 16 lane Roche 454 FLX Titanium run. Coupled with robust bioinformatics approaches, enhancements in NGS workflow will generate a framework for executing large-scale and realistic pilot projects in biomonitoring and related applications and provide insights for analyzing various bulk environmental samples in a similar manner.

## Methods

### Specimen collection and handling

A larval benthic sample was collected from the Humber River (43º54^′^12.88″N, 79º42^′^35.61″W). The sample was collected by kick netting for ten transects across the river, 10 m between transects. The sample was live sorted on site and preserved in 95% ethanol at 4°C until processing (approximately 3 months).

### Construction of Sanger DNA barcode library

From the bulk sample, all individuals were sorted into 96-well plates and morphologically identified to the order level. A single leg from each individual (about 2 mm fragment size) was then subjected to routine DNA barcoding following standard COI DNA barcoding protocols [14]. We amplified standard full-length (~650 bp) COI DNA barcodes with LCO1490/HCO2198 primers [15] using a standard pre-made PCR mixture followed by standard Sanger sequencing in an Applied Biosystems 3730XL DNA sequencer [14]. All sequences have been submitted to GenBank (Accession Numbers KC263052 - KC263197).

### DNA extraction from bulk environmental sample

We performed two DNA preparations for NGS analysis. One method involved using ethanol originally used for collecting and preserving benthic sample (see above). For this ethanol-based DNA analysis, 10 ml of the preserved ethanol was transferred to ten 1.5 ml Eppendorf tubes and evaporated at 56°C for 5–6 hours. The dried residue was dissolved in 30 μl of molecular biology grade water and was used as the source of template DNA for PCR (see below).

The other approach involved using tissue mixtures from the original benthic sample. A tissue sample from each individual (e.g. a leg; about 2 mm fragment size) was pooled in a single tube containing ethanol and the resultant mixed tissue sample was incubated at 56°C for approximately two hours to evaporate residual ethanol. The dried mixture was divided into 10 lysing matrix tubes “A” (about 100 mg each) and homogenized using an MP FastPrep-24 Instrument (MP Biomedicals Inc.) at speed 6 for 40 sec. Total DNA of this homogenized slurry was extracted using the Nucleospin tissue kit (Macherey-Nagel Inc.) following the manufacturer’s instructions and eluted in 50 μl of molecular biology grade water.

### PCR amplification conditions

Three fragments within the standard COI DNA barcode region were amplified with three newly designed primer sets (Table 2) in a two-step PCR amplification regime [4]. The first PCR used COI specific primers and the second PCR involved 454 fusion-tailed primes. In the first PCR, ten amplicons (for each primer set) were generated from both ethanol-based DNA and tissue-DNA. The PCR reactions were assembled in 25 μl volumes. Each reaction contained 2 μl DNA template, 17.5 μl molecular biology grade water, 2.5 μl 10× reaction buffer (200 mM Tris–HCl, 500 mM KCl, pH 8.4), 1 μl MgCl_2_ (50 mM), 0.5 μl dNTPs mix (10 mM), 0.5 μl forward primer (10 mM), 0.5 μl reverse primer (10 mM), and 0.5 μl Invitrogen’s Platinum Taq polymerase (5 U/μl). The PCR conditions were initiated with heated lid at 95°C for 5 min, followed by a total of 15 cycles of 94°C for 40 sec, 46°C for 1 min, and 72°C for 30 sec, and a final extension at 72°C for 5 min, and hold at 4°C. Amplicons from each sample were pooled and purified using Qiagen’s MiniElute PCR purification columns and eluted in 30 μl molecular biology grade water. The purified amplicons from the first PCR were used as templates in a second PCR with the same amplification condition used in the first PCR with the exception of using 454 fusion-tailed primers in a 30-cycle amplification regime. Eppendorf Mastercycler ep gradient S thermalcyclers were used for all PCRs. A negative control reaction (no DNA template) was included in all experiments. PCR success was checked by agarose gel electrophoresis.

**Table 2:** PCR primer sets used for amplification of three fragments in COI DNA barcode region

**Primer set**	**Sequence** (**5**^′^-**3**^′^)	**Amplicon size(bp)**
**AD**	Forward: GGIGGITTTGGIAATTGAYTIGTICC	191
	Reverse: CCTARIATIGAIGARAYICCIGC	
**BE**	Forward: CCIGAYATRGCITTYCCICG	224
	Reverse: GTRATIGCICCIGCIARIAC	
**CF**	Forward: GITGAACIGTITAYCCICC	197
	Reverse: CCIGCIGGRTCIAARAAIGAIGT	

### 454 Pyrosequencing

Amplicons were quantified by flourometer and normalized to the same concentration (100 ng/μl). The amplicon libraries were sequenced on a 454 Genome Sequencer FLX System (Roche Diagnostics GmbH) following the amplicon sequencing protocol and using GS Titanium chemistry. Amplicons of each sample were bi-directionally sequenced in 1/16 of a full sequencing run (70 × 75 picotiter plate). Details of the 454 pyrosequencing run are available by request from the corresponding author. All sequences have been submitted to GenBank (Accession Numbers KC263198 - KC282326).

### Data analysis

Pyrosequencing reads were first filtered by quality using ‘Filter FASTQ’ on Galaxy [16-18] ensuring that 90% of bases in each read were assigned a Phred score above 15. Sequences originating from each primer set were separated, allowing for one mismatch within the primer region. The reference database was compiled from a Sanger library with taxonomy determined by BOLD v2.5 [19]. The reference database was dereplicated using the USEARCH algorithm [20]. Comparisons of pyrosequences and Sanger sequences were performed using a stand-alone version of blastn [21] with a minimum id of 98% and bit score of 100. Sequences which did not produce blast hits were queried against the same reference library at a less stringent (95%) identity using megablast [22].

A total of 21932 sequences were generated in one lane, and 23483 in the other using DNA templates from ethanol-based DNA and tissue-DNA, respectively. After quality filtering, there were 17308 (~79%) reads and 20240 (~86%) for each lane, respectively. The Sanger reference library contained a total of 148 sequences, but after clustering with USEARCH [20] with a 98% identity, it was reduced to 46 representative OTUs. The tree was constructed using the Neighbor-Joining method [23] with distances calculated using the number of differences method with the software package MEGA5 [24].

## Competing interests

The authors declare that they have no competing interests.

## Authors’ contributions

MH conceived the idea, designed the experiments, participated in data analysis and visualization, and wrote the manuscript. JLS collected the benthic sample, performed molecular biology analysis, participated in data analysis and edited the manuscript. SS participated in experimental design, performed NGS DNA sequencing, participated in data analysis, and edited the manuscript. SVK performed bioinformatics analyses of DNA sequence data. All authors read and approved the final manuscript.

## Supplementary Material

Additional file 1 **Table S1.** Taxa identified from sequences obtained in environmental barcoding analysis but absent in Sanger sequenced DNA barcode library constructed from individuals in the benthic sample analysed. Click here for file

## References

[B1] Margulies M Egholm M Altman WE Attiya S Bader JS Bemben LA Berka J Braverman MS Chen YJ Chen Z Genome sequencing in microfabricated high-density picolitre reactors Nature 2005 437 7057 376 380 16056220 10.1038/nature03959 PMC1464427

[B2] Sogin ML Morrison HG Huber JA Welch DM Huse SM Neal PR Arrieta JM Herndl GJ Microbial diversity in the deep sea and the underexplored “rare biosphere” Proc Natl Acad Sci U S A 2006 103 32 12115 12120 10.1073/pnas.0605127103 16880384 PMC1524930

[B3] Taberlet P Coissac E Hajibabaei M Rieseberg LH Environmental DNA Mol Ecol 2012 21 8 1789 1793 10.1111/j.1365-294X.2012.05542.x 22486819

[B4] Hajibabaei M Shokralla S Zhou X Singer GA Baird DJ Environmental barcoding: a next-generation sequencing approach for biomonitoring applications using river benthos PLoS One 2011 6 4 e17497 10.1371/journal.pone.0017497 21533287 PMC3076369

[B5] Bonada N Prat N Resh VH Statzner B Developments in aquatic insect biomonitoring: a comparative analysis of recent approaches Annu Rev Entomol 2006 51 495 523 10.1146/annurev.ento.51.110104.151124 16332221

[B6] Baird DJ Hajibabaei M Biomonitoring 2.0: a new paradigm in ecosystem assessment made possible by next-generation DNA sequencing Mol Ecol 2012 in press 10.1111/j.1365-294x.2012.05519.x 22590728

[B7] Shokralla S Singer GA Hajibabaei M Direct PCR amplification and sequencing of specimens’ DNA from preservative ethanol Biotechniques 2010 48 3 233 234 10.2144/000113362 20359306

[B8] Polz MF Cavanaugh CM Bias in template-to-product ratios in multitemplate PCR Appl Environ Microbiol 1998 64 10 3724 3730 9758791 10.1128/aem.64.10.3724-3730.1998 PMC106531

[B9] Shokralla S Spall JL Gibson JF Hajibabaei M Next-generation sequencing technologies for environmental DNA research Mol Ecol 2012 21 1794 1805 10.1111/j.1365-294X.2012.05538.x 22486820

[B10] Ficetola GF Miaud C Pompanon F Taberlet P Species detection using environmental DNA from water samples Biol Lett 2008 4 4 423 425 10.1098/rsbl.2008.0118 18400683 PMC2610135

[B11] Taberlet P Prud’homme SM Campione E Roy J Miquel C Shehzad W Gielly L Rioux D Choler P Clement JC Soil sampling and isolation of extracellular DNA from large amount of starting material suitable for metabarcoding studies Mol Ecol 2012 21 8 1816 1820 10.1111/j.1365-294X.2011.05317.x 22300434

[B12] Creer S Fonseca VG Porazinska DL Giblin-Davis RM Sung W Power DM Packer M Carvalho GR Blaxter ML Lambshead PJ Ultrasequencing of the meiofaunal biosphere: practice, pitfalls and promises Mol Ecol 2010 19 Suppl 1 4 20 20331766 10.1111/j.1365-294X.2009.04473.x

[B13] Ranasinghe JA Stein ED Miller PE Weisberg SB Performance of two southern california benthic community condition indices using species abundance and presence-only data: relevance to DNA barcoding PLoS One 2012 7 8 e40875 10.1371/journal.pone.0040875 22879881 PMC3413687

[B14] Hajibabaei M deWaard JR Ivanova NV Ratnasingham S Dooh RT Kirk SL Mackie PM Hebert PDN Critical factors for assembling a high volume of DNA barcodes Philos Trans R Soc Lond B Biol Sci 2005 360 1462 1959 1967 10.1098/rstb.2005.1727 16214753 PMC1609220

[B15] Folmer O Black M Hoeh W Lutz R Vrijenhoek R DNA primers for amplification of mitochondrial cytochrome c oxidase subunit I from diverse metazoan invertebrates Mol Mar Biol Biotechnol 1994 3 5 294 299 7881515

[B16] Blankenberg D Von Kuster G Coraor N Ananda G Lazarus R Mangan M Nekrutenko A Taylor J Ausubel FM Galaxy: a web-based genome analysis tool for experimentalists Current protocols in molecular biology 2010 11 21 Chapter 19:Unit 19 10 10.1002/0471142727.mb1910s89 PMC4264107 20069535

[B17] Giardine B Riemer C Hardison RC Burhans R Elnitski L Shah P Zhang Y Blankenberg D Albert I Taylor J Galaxy: a platform for interactive large-scale genome analysis Genome Res 2005 15 10 1451 1455 10.1101/gr.4086505 16169926 PMC1240089

[B18] Goecks J Nekrutenko A Taylor J Galaxy: a comprehensive approach for supporting accessible, reproducible, and transparent computational research in the life sciences Genome Biol 2010 11 8 R86 10.1186/gb-2010-11-8-r86 20738864 PMC2945788

[B19] Ratnasingham S Hebert PDN BOLD: the Barcode of Life Data System ( http://www.barcodinglife.org) Mol Ecol Notes 2007 7 355 364 10.1111/j.1471-8286.2007.01678.x 18784790 PMC1890991

[B20] Edgar RC Search and clustering orders of magnitude faster than BLAST Bioinformatics 2010 26 19 2460 2461 10.1093/bioinformatics/btq461 20709691

[B21] Altschul SF Gish W Miller W Myers EW Lipman DJ Basic local alignment search tool J Mol Biol 1990 215 3 403 410 2231712 10.1016/S0022-2836(05)80360-2

[B22] Morgulis A Coulouris G Raytselis Y Madden TL Agarwala R Schaffer AA Database indexing for production MegaBLAST searches Bioinformatics 2008 24 16 1757 1764 10.1093/bioinformatics/btn322 18567917 PMC2696921

[B23] Saitou N Nei M The neighbor-joining method: a new method for reconstructing phylogenetic trees Mol Biol Evol 1987 4 4 406 425 3447015 10.1093/oxfordjournals.molbev.a040454

[B24] Tamura K Peterson D Peterson N Stecher G Nei M Kumar S MEGA5: molecular evolutionary genetics analysis using maximum likelihood, evolutionary distance, and maximum parsimony methods Mol Biol Evol 2011 28 10 2731 2739 10.1093/molbev/msr121 21546353 PMC3203626

