# Supplementary material for: Assessing biodiversity of a freshwater benthic macroinvertebrate community through non-destructive environmental barcoding of DNA from preservative ethanol
Source: BMC Ecol. 2012 Dec 23;12:28. doi: 10.1186/1472-6785-12-28 (PMC3542036; doi:10.1186/1472-6785-12-28)
Supplement: Additional file 1 — Table S1. Taxa identified from sequences obtained in environmental barcoding analysis but absent in Sanger sequenced DNA barcode library constructed from individuals in the benthic sample analysed. [file 1472-6785-12-28-S1.doc]

APPENDIX 1

**Table 1.** OTUs identified from sequences obtained in environmental barcoding analysis but absent in Sanger sequenced DNA barcode library constructed from individuals in the benthic sample analysed.

| **Taxon** | **DNA source-Primer set**  **(# 454 reads obtained)** | | | | | |
| --- | --- | --- | --- | --- | --- | --- |
|  | **E-AD** | **T-AD** | **E-BE** | **T-BE** | **E-CF** | **T-CF** |
| Trichoptera, *Hydropsyche slossonae* |  |  | 2 |  | 38 | 54 |
| Trichoptera, Hydropsychidae sp. |  |  | 1 | 4 |  |  |
| Trichoptera, *Cheumatopsyche* sp. |  |  | 7 |  |  |  |
| Diptera, *Simulium tuberosum* | 2 | 3 | 23 | 24 | 15 | 20 |
| Diptera, *Dicranota* sp. |  |  | 3 | 1 | 1 | 2 |
| Diptera, Chironomidae sp. |  | 1 | 3 |  |  |  |
| Plecoptera, *Leuctra rickeri* |  |  |  | 1 | 2 | 1 |
| Plecoptera, *Leuctra* sp. |  |  | 1 | 3 |  |  |
| Primates, *Homo sapiens* |  |  | 14 |  |  |  |
| Trombidiformes, *Lebertia* sp. |  |  | 23 |  |  |  |
| Haplotaxida, Naididae sp. |  |  | 8 |  |  |  |
| Malpighiales, *Populus balsamifera* |  |  | 3 |  |  |  |
| Ephemeroptera, *Baetis tricaudatus* |  |  | 1 |  |  |  |
| Ephemeroptera, Leptophlebiidae sp. | 3 | 1 | 5 | 3 |  | 1 |
| Ephemeroptera, *Ephemerella* sp. |  | 1 |  |  |  |  |
| Carnivora, Canidae, *Canis familiaris/lupus* |  | 1 |  |  |  |  |

E=Ethanol-based DNA, T=Tissue-DNA
